# Expression of the immune checkpoint receptor TIGIT in Hodgkin’s lymphoma

**DOI:** 10.1186/s12885-018-5111-1

**Published:** 2018-12-04

**Authors:** Wenchao Li, Niclas C. Blessin, Ronald Simon, Martina Kluth, Kristine Fischer, Claudia Hube-Magg, Georgia Makrypidi-Fraune, Björn Wellge, Tim Mandelkow, Nicolaus F. Debatin, Laura Pott, Doris Höflmayer, Maximilian Lennartz, Guido Sauter, Jakob R. Izbicki, Sarah Minner, Franziska Büscheck, Ria Uhlig, David Dum, Till Krech, Andreas M. Luebke, Corinna Wittmer, Frank Jacobsen, Eike Burandt, Stefan Steurer, Waldemar Wilczak, Andrea Hinsch

**Affiliations:** 1 Department of Pathology, University Medical Center Hamburg-Eppendorf, Martinistr. 52, 20246 Hamburg, Germany; 2 Dianova GmbH, Warburgstrasse 45, 20354 Hamburg, Germany; 3 Department of General, Visceral and Thoracic Surgery, University Medical Center Hamburg-Eppendorf, Hamburg, Germany

**Keywords:** TIGIT, PD-1, Immune checkpoint, Hodgkin’s lymphoma

## Abstract

Hodgkin’s lymphoma (HL) is characterized by a high background of inflammatory cells which play an important role for the pathogenesis of the disease. T cell immunoreceptor with Ig and ITIM domains (TIGIT) is an inhibitory immune checkpoint receptor and a putative target for novel immunotherapies. To study patterns of TIGIT expression in the T cell background surrounding malignant cells including Hodgkin cells, Reed-Sternberg cells and histiocytic cells, a microenvironment (ME) tissue microarray (TMA) was constructed from tissue punches measuring 2 mm in diameter obtained from formalin-fixed tissue samples of Hodgkin’s lymphoma lymph nodes (*n* = 40) and normal human tonsil (*n* = 2). The ME-TMA was stained by brightfield and fluorescence multiplex immunohistochemistry (IHC) to evaluate expression levels of TIGIT and PD-1 as well as standard lymphocyte markers (CD3, CD8, CD4, FOXP3) in the lymphocytic background. All analyzed cases of HL contained 9–99% (median: 86%) of TIGIT^+^ lymphoid cells. In general, TIGIT localized to the same cells as PD-1. Strikingly, expression levels of TIGIT and PD-1 were highly variable among the analyzed samples. Highest levels of TIGIT and PD-1 were found in one sample of nodular lymphocytic-predominant HL (NLPHL). In conclusion, TIGIT expression is highly variable between patients with Hodgkin’s lymphoma. Our results encourage further studies evaluating the role of TIGIT as a target for immunotherapies in Hodgkin’s lymphoma.

**Electronic supplementary material:**

The online version of this article (10.1186/s12885-018-5111-1) contains supplementary material, which is available to authorized users.

## Introduction

Hodgkin’s lymphoma (HL) is a malignant transformation of B cell origin that accounts for about 20–30% of lymphomas in Western societies [1–3]. Although the majority of Hodgkin’s lymphoma patients are curable with a multi-agent chemotherapy and/ or radiotherapy protocol, about 10–20% of patients develop therapy refractory disease [4]. A characteristic feature of HL is the presence of few malignant cells, including Hodgkin cells, Reed-Sternberg cells and histiocytic cells, in a high background of inflammatory cells. Current clinical trials provide first evidence that immune checkpoint inhibitors targeting the PD-1/PD-L1 axis may hold promise in these refractory or relapsed Hodgkin’s lymphomas [5–7].

T cell immunoglobulin and ITIM domain (TIGIT), a co-inhibitory transmembrane glycoprotein of the poliovirus receptor (PVR)/−nectin superfamily, is another interesting candidate for novel checkpoint therapies [8, 9]. Using multiplex fluorescence immunohistochemistry, we have recently shown that TIGIT typically co-localizes with PD-1 on CD8+ cytotoxic T cells, CD4+ T helper cells and FOXP3 regulatory T cells [10]. Tumor associated lymphocytes expressing TIGIT have so far been demonstrated in acute myeloid leukemia, non-small cell lung cancer, colorectal carcinoma and melanoma [11–13]. Although the downstream signaling cascade of TIGIT has not been clarified, there is evidence that TIGIT negatively regulates T cell activity through downregulation of T cell receptor expression [8, 14, 15]. In mouse models and ongoing clinical studies, blockade or ablation of TIGIT, alone or in combination with blockade of programmed cell death protein (PD-1), can restore tumor suppressive effects [11, 12, 16–18]. These findings indicate that TIGIT, similar to PD-1, has a crucial role in inhibiting the tumor-directed immune response and, thus, might be a suitable and relevant target for novel immune-modulating therapies. Several drugs targeting TIGIT are currently under development [19].

As to yet, data on the possible role of TIGIT in Hodgkin’s lymphoma are lacking. Here, we made use of a microenvironment (ME) tissue microarray (TMA) that was constructed from 2 mm tissue punches each from 40 Hodgkin’s lymphomas and studied patterns of TIGIT and PD-1 expression by means of conventional bright field and multiplex fluorescence immunohistochemistry.

## Material and methods

### Tissues

Formalin-fixed paraffin-embedded tissue samples from 40 patients with Hodgkin’s lymphoma were selected from the archives of the institute of Pathology of the University Medical Center Hamburg-Eppendorf, Germany. The selection included 30 patients diagnosed with nodular sclerosis classical HL (NSCHL), 7 patients with mixed cellularity classical HL (MCCHL), one patient with lymphocyte rich classical HL (LRCHL), and one patient with nodular lymphocyte predominance HL (NLPHL). The histological subtype was undetermined for another patient.

### Microenvironment (ME) TMA construction

A pathologist reviewed all cases and selected areas containing Hodgkin and Reed-Sternberg cells for TMA construction. A single tissue punch measuring 2 mm in diameter was taken from each donor tissue block to capture the lymphocytic background adjacent to the malignant cells. In addition, two punches of normal human tonsil were added to the TMA as a reference tissue for fluorescence measurement normalization. The usage of archived diagnostic left-over tissues for manufacturing of tissue microarrays and their analysis for research purposes as well as patient data analysis has been approved by local laws (HmbKHG, §12,1) and by the local ethics committee (Ethics commission Hamburg, WF-049/09).

### Immunohistochemistry

Freshly cut 4 μm tissue sections were used for immunohistochemistry (IHC) analyses. For brightfield IHC, tissue sections were dewaxed and incubated in an autoclave for 5 min at 121 °C in Tris-EDTA pH 7.8 antigen retrieval solution prior to blocking of endogenous peroxidase and incubation of the primary antibody (Dianova mouse anti TIGIT, clone TG-1,1:70). Bound antibody was detected with the DAB-kit (DAKO, Santa Clara, United States) and slides were counterstained and sealed in EUKITT®. For fluorescence multiplex IHC, the Opal™ dye kit (Cat. #OP7DS1001KT, Perkin Elmer, Waltham, Massachusetts, United States) was used. The experimental procedure was performed according to the manufacturer’s instructions. Slides were initially boiled in a microwave oven for 15 min at 100 °C for antigen retrieval. Three different primary antibodies were combined with DAPI staining in each experiment. One circle of antibody staining included peroxidase blocking, application of the primary antibody, detection with a secondary HRP-conjugated antibody, fluorescence dye detection, and removal of the bound antibodies by microwave treatment (15 min at 100 °C). This cycle was repeated two times for the remaining antibodies. Slides were subsequently counterstained with diamidino-2-phenylindole (DAPI) and mounted in antifade solution. Details on the used antibodies, antibody retrieval procedures and Opal™ dyes are given in Table 1.

**Table 1:** List of the used antibodies, antigen retrieval (AR), dilutions and Opal™ dyes

		Bright field		Fluorescence			
Antibody	Target	AR	Dilution	AR	Dilution	Order^1^	Dye
DAKO #IR503	CD3	pH 9	1:1	pH 9	1:1	1st	Opal 520
DAKO #IR649	CD4	pH 9	1:1	pH 9	1:1	1st	Opal 520
DAKO #IR623	CD8	pH 9	1:1	pH 9	1:1	1st^a^	Opal 520^b^
BioLegend #320102	FOXP3	pH 9	1:50	pH 9	1:50	1st	Opal 520
DAKO #IR604	CD20	pH 9	1:1	pH 9	1:10	1st^a^	Opal 520^b^
Dianova #DIA-TG1	TIGIT	pH 7.8	1:70	pH 9	1:150	2nd	Opal 570
Abcam #ab52587	PD-1	pH 6	1:50	pH 6	1:50	3rd	Opal 690

Order^1^, order refers to the sequence of antibodies in multiplex fluorescence immunohistochemistry experiments

^a^antibody was used at third position when stained in combination with CD3, CD4

^b^with Opal™ 690 dye

### Quantification of TIGIT and PD-1 expression

Digital images of multiplex immunofluorescence stainings were acquired using a Leica Aperio VERSA 8 automated epifluorescence microscope. For initial image acquisition, exposure time was manually adjusted for each fluorochrome to minimize auto fluorescence. Subsequently, a threshold for positive staining was defined as follows: The fluorescence intensity of each antibody was measured in 50 to 200 cells of a cell type with known lack of expression and the fluorescence value of the cell with highest “false positive” measurement was used to define the cutoff value for positive expression.

After slide scanning, image analysis was performed using Image Scope software package (Leica Microsystems Wetzlar, Germany). Image analysis included segmentation of individual cells and subsequent measurement of the fluorescence intensity (concentration as dye intensity per μm^2^). Tissue areas for analysis were manually defined and typically included between 18,000 and 30,000 cells. Fluorescence intensity values were recorded for each fluorochrome in each cell. The average fluorescence intensity of all cells of the same compartment from all measured areas was calculated and used for comparisons. Because staining intensity largely depends on antibody specification and staining protocols we sought to introduce a reference tissue for staining normalization to our experiments. We selected two samples of tonsil tissue, which were placed on the same TMA along with the study samples. In our previous study [10], we found that cells located within the germinal centers of tonsil tissues had the highest TIGIT and PD-1 expression level of all human normal tissues. We calculated an average from approximately 1000 lymphocytes located at the periphery of the germinal centers (500 from each tonsil), considered this expression level as 100% and used it as the reference value to estimate the staining intensity of TIGIT and PD-1 on CD3 positive cells in the lymphocytic background of HL (relative expression, RE). In addition, the expression level of CD3 was used as an immunoreactivity control to indicate potential fixation or processing artefacts.

### Statistics

JMP Pro 12 software package (SAS Institute Inc., NC, USA) analysis of variance (ANOVA) test was employed to calculate compartment specific expression differences of PD-1 and TIGIT in tissue samples.

## Results

### TIGIT brightfield immunohistochemistry

**Fig. 1:**
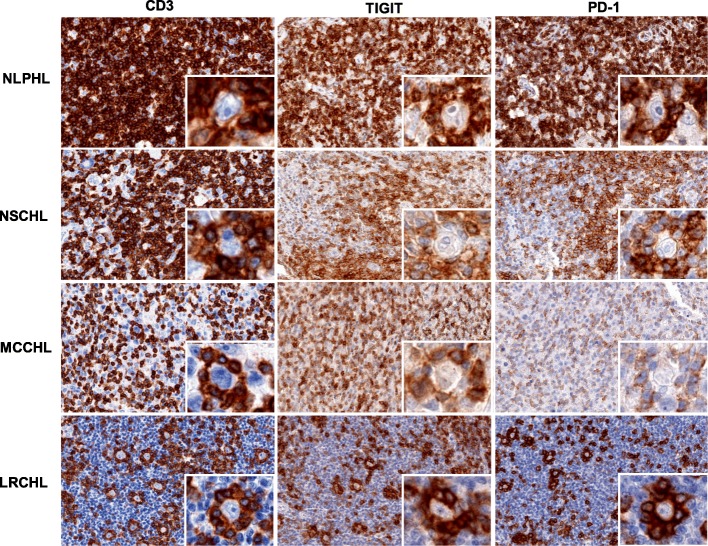
Representative images of CD3, TIGIT and PD-1 staining in nodular lymphocyte predominance HL (NLPHL), nodular sclerosis classical HL (NSCHL), mixed cellularity classical HL (MCCHL) and lymphocyte rich classical HL (LRCHL). Insets show magnifications of Hodgkin and Reed-Sternberg cells surrounded by TIGIT and PD-1 expressing CD3 positive T cells

All 40 HL included in this study were successfully analyzed and showed detectable TIGIT staining in 9–99% (Median: 86%) of the lymphocytic background cells by means of conventional bright field immunohistochemistry. Representative images are shown in Fig. 1. By manual inspection of the stained slide, highest staining intensities were found in a case of NLPHL. Additional experiments with serial dilutions of the TIGIT antibody confirmed the presence of T cells with extraordinary high levels of TIGIT expression (i.e. exceeding that of normal human tonsils germinal centers) particularly in NLPHL (Additional file 1: Figure S1). Also, staining intensity of the T cell rosettes surrounding malignant (HRS and LP) cells in NLPHL and in LRCHL appeared somewhat stronger as compared to scattered CD3 positive lymphocytes in the vicinity (Fig. 1).

### Multiplex fluorescence immunohistochemistry

**Fig. 2:**
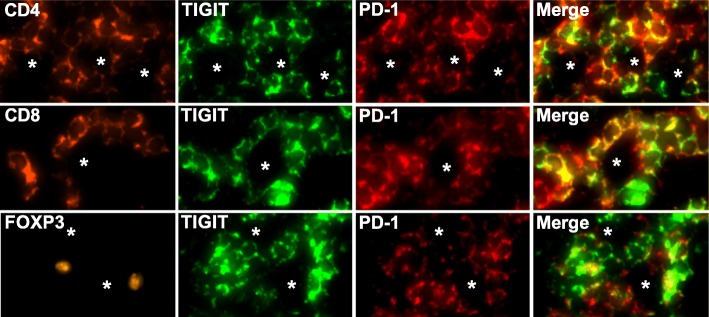
Representative multiplex immunofluorescence images showing TIGIT and PD-1 staining in CD4 positive T helper cells (top panel), CD8 positive cytotoxic T cells (middle panel) and FOXP3 regulatory T cells (bottom panel)

**Fig. 3:**
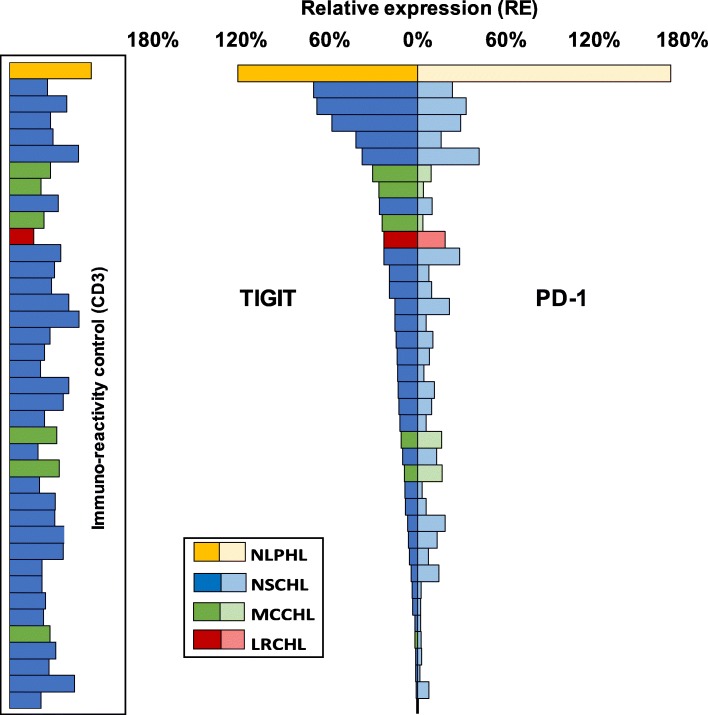
Variability of TIGIT (left) and PD-1 (right) expression in CD3 positive T cells in the lymphocytic background of different types of Hodgkin’s lymphoma (HL). Relative expression refers to the fluorescence measurement in normal human tonsils germinal centers set to 100%. NLPHL, nodular lymphocyte predominance HL; NSCHL, nodular sclerosis classical HL; MCCHL, mixed cellularity classical HL; LRCHL, lymphocyte rich classical HL. Bars in the box indicate expression levels of CD3 as an indicator of immunoreactivity of the analyzed tissue samples

We performed multiplex fluorescence immunohistochemistry to unravel the immune cell types expressing TIGIT and to quantify TIGIT expression among individual patients. TIGIT was virtually undetectable on CD20 positive B lymphocytes but expressed on a large fraction (70%) of CD3 positive T lymphocytes. Further analysis revealed that TIGIT positive T cell subtypes included CD8 cytotoxic T cell, CD4 helper T cells and FOXP3 regulatory T cells (Fig. 2). Interestingly, relative expression (RE) levels of TIGIT were highly variable among individual patients and different HL subtypes. When compared to the RE level found in the reference tissue (normal human tonsil germinal center periphery set to 100% RE), TIGIT RE in CD3 positive T cells ranged between 1 and 122% in different patients. Most patients had TIGIT expression levels equaling less than 50% of the reference tissue. Again, highest TIGIT expression (122%) was seen in one case of nodular lymphocyte-predominant HL (NLPHL, Fig. 3).

### Relationship between TIGIT and PD-1 expression

**Fig. 4:**
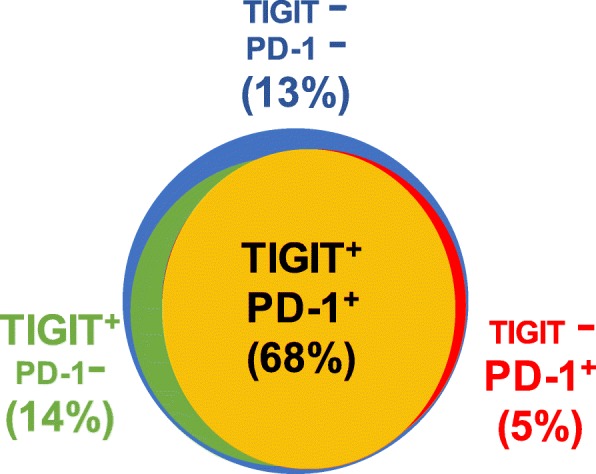
Average fractions of CD3 positive T cells expressing TIGIT only (green), PD-1 only (red), both (yellow) or neither one (blue) in 30 cases of nodular lymphocyte predominance HL (NSCHL)

To estimate the fraction of CD3 positive T cells expressing TIGIT only, PD-1 only, both, or none of the two, we focused on the largest HL subtype (*n* = 30 NSCHL). On average across all patients, it showed that the majority (68%) of T cells had co-expression of TIGIT and PD-1, while isolated positivity for TIGIT and PD-1 was seen in only 14 and 5%. The remaining 13% T cells had neither TIGIT nor PD-1 expression (Fig. 4). Fluorescence measurements further confirmed presence of T cells with very high levels of TIGIT and PD-1 expression in NLPHL. For example, the top 10 % of TIGIT and PD-1 positive T cells averaged 241% RE and 408% RE, respectively.

## Discussion

The data from this study demonstrate that TIGIT is frequently expressed in cells of the lymphocytic background accompanying HL and that the frequency and intensity of TIGIT expression varies markedly between individual patients.

All 40 HL analyzed in our study showed detectable TIGIT expression in all types of CD3 positive T cells including CD8 cytotoxic T cells, CD4 positive T helper cells, and FOXP3 positive regulatory T cells. This corresponds to the situation in other normal and inflamed tissues, where we also found that TIGIT expression was largely limited to these three major types of immune cells. In this earlier study, we also found that TIGIT expression often paralleled PD-1 expression in these cells. It was therefore expected that a large fraction (more than 80%) of TIGIT positive T cells also expressed PD-1 in our study. In normal lymph nodes and tonsils, TIGIT and PD-1 co-expression was found in 50–99% of T cells depending on the analyzed T cell subtype [10]. These findings are in line with a study describing that TIGIT (and PD-1) are constitutively expressed on about one third of circulating FOXP3 positive regulatory T cells and highly upregulated on regulatory T cells in an inflamed microenvironment [20, 21]. This suggests that the lymphocytic background of HL is affected by the same immunoregulatory mechanisms that also apply in inflammatory and cancerous tissues.

In contrast to many other studies that reported PD-1 staining in 20–60% of classical HL [22–27], we detected PD-1 positive lymphocytes in all analyzed cases. Some of the discrepant findings may be related to different definitions of PD-1 positivity in earlier work, for example studies where PD-1 positivity was assumed only when more than 20% of cells [27] or more than 23 cells/mm^2^ [26] had detectable PD-1 staining. While it is obviously impossible to explain all discrepant reports, we previously found that certain levels of TIGIT and PD-1 expression are characteristic features of lymphatic infiltrations in a wide range of inflamed and cancerous tissue [10].

The fluorescence approach enabled us to quantify the expression level of TIGIT and PD-1. We have recently shown that highest physiological TIGIT and PD-1 expression was found in follicular T helper cells residing at the periphery of germinal centers in lymph nodes and tonsils [10]. Given this consistently high expression levels and because of the easiness to identify follicular T cells, we have used these cells as a reference to compare the variable TIGIT and PD-1 expression of T cells located in other tissue areas. Accordingly, all measured intensity levels that were lower than 100% do, however, not imply reduced TIGIT and PD-1 expression of the respective cells. We recorded a stunning degree of TIGIT and PD-1 expression level variability across the different HL specimens analyzed. The much less variable expression levels of CD3 in our cancers argue against relevant fixation related variations in tissue immunoreactivity as a possible reason for the observed interindividual TIGIT expression differences. Biological reasons for the variable expression might include modulation of the immune environment by the Hodgkin and Reed-Sternberg (HRS) cells or the lymphocytic background itself. For example, it has been suggested that the expression of PD-1 on T cells is likely driven by constitutive upregulation of its ligands, PD-L1 and PD-L2, on HRS cells [28] and that HRS cells as well as some leukocytes (e.g. helper and regulatory T cells or macrophages) [29] can selectively express immunomodulatory proteins connected to PD-1 signaling such as galectin 1 and IL 10 [30, 31]. Hence, it is tempting to speculate that also the level of TIGIT expression may be influenced by regulatory molecules in a similar way as known from PD-1.

Notably, our series of HL included one case of nodular lymphocyte predominant Hodgkin’s lymphoma (NLPHL). A unique feature of NLPHL is that the lymphocytic predominant (LP) cells (previously known as “popcorn” cells) arise in a follicular like microenvironment that shares characteristics of germinal centers in lymph nodes and tonsils, such as a high density of follicular T helper cells [32, 33]. That highest TIGIT and PD-1 levels in our current study were also found in a HL subtype with a characteristic follicular architecture suggests an important function of these receptors in dense meshworks of leucocytes. Highest levels of TIGIT and PD-1 in tightly packed lymphocytic rosettes surrounding the neoplastic cells fits well to the concept of compensatory downregulation of excessive inflammatory reactions through immune checkpoint upregulation [34, 35]. Exceedingly high PD-1 expression levels in NLPHL has earlier been described and PD-1 expression analysis was suggested as a diagnostic feature for this rare HL subtype [36]. Our fluorescent measurement revealed that the TIGIT and PD-1 expression in individual T cells could reach 2 to 4-fold higher levels than those found in normal human tonsils.

Another feature of HL is that the tumor cells have developed mechanisms to escape the excessive immune-cell infiltration of the host. For example, the cellular landscape of HL is characterized by a paucity of specific cytotoxic T cells and natural killer (NK) cells [37]. Much hope is, therefore, put on novel therapeutic strategies modulating the immune system and its response to the disease, particularly in heavily treated relapsed or refractory HL where effective therapies are lacking. After promising results were reported from a first clinical trial [5] using the PD-1 inhibitor nivolumab in chemotherapy refractory HL, a number of follow up studies have confirmed the potential of immune checkpoint therapy in this disease [38–41]. Phase I/II studies using nivolumab as a combination or second line therapy in combination with or after anti-CD30 treatment in patients with relapsed and refractory Hodgkin’s lymphoma reported over all response rates of 66–85% [38, 39]. Combining immunotherapies is another option to increase response rates. For example, combination of anti-PD-1 and anti-CTLA4 drugs has demonstrated additive efficacy in melanomas, non-small-cell lung cancer and renal cell carcinoma [6, 42, 43]. At present, several drugs directed against TIGIT are in preclinical pipelines [17, 19]. In our study, TIGIT and PD-1 expression was found in all analyzed HL and was not limited to a specific subtype. These findings identify HL as a disease entity where it could be interesting to determine whether TIGIT inhibitors alone or in combination with other immunotherapies and anti-CD30 treatment might be effective. It might be speculated, that the variable levels of TIGIT expression could correspond to variable responses to putative therapies targeting TIGIT.

The large tissue size TMA (ME-TMAs) enable the analysis for large enough tissue areas to study a tumor’s microenvironment under perfectly standardized experimental conditions. Having all tissue samples of a study on one glass slide ensures not only fully identical staining procedures for every tissue specimen but also identical section thickness and slide age [44]. This is important for experimental standardization as automated immunostainers are not yet perfectly dealing with the requirements of multiplex fluorescence immunohistochemistry.

In summary, the results of our study identify HL as a tumor type with frequent but variable expression of immune checkpoint receptors such as TIGIT and PD-1. Patients with HL might benefit from potential future therapies targeting TIGIT alone or in combination with other drugs.

## Additional file

Additional file 1: **Figure S1.** Serial dilution of the TIGIT antibody in lymph node and a NLPHL. (PDF 6352 kb)
