# Supplementary figures and images for: Expression of the immune checkpoint receptor TIGIT in Hodgkin’s lymphoma
Source: BMC Cancer. 2018 Dec 4;18:1209. doi: 10.1186/s12885-018-5111-1 (PMC6280445; doi:10.1186/s12885-018-5111-1)

## Tonsilla palatina

1:70

TIGIT

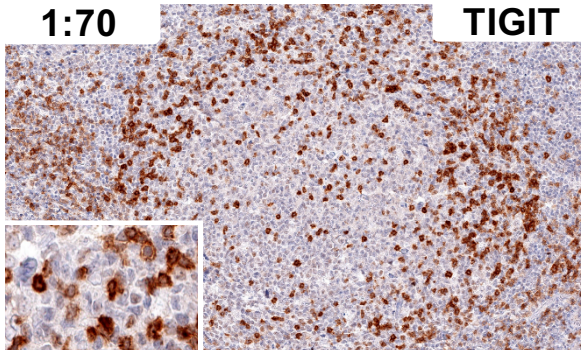

1:210

TIGIT

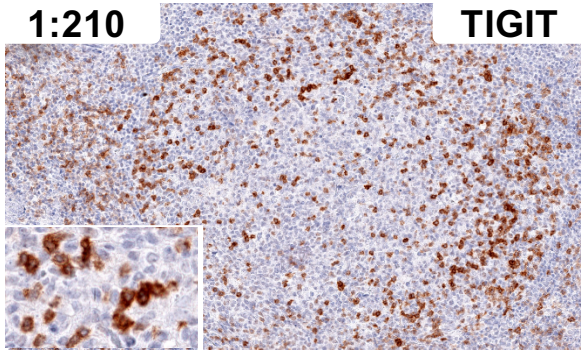

1:630

TIGIT

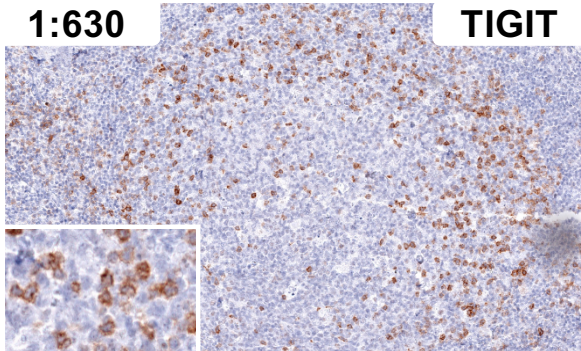

1:1890

TIGIT

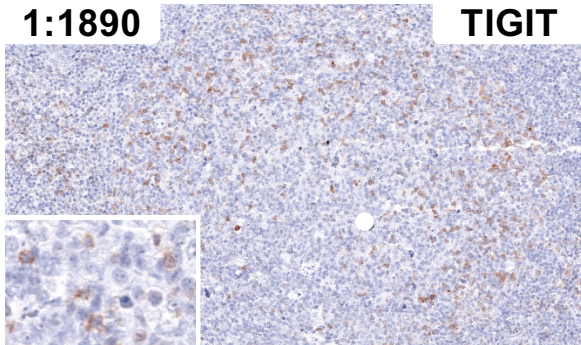

## Hodgkin lymphoma

1:70

TIGIT

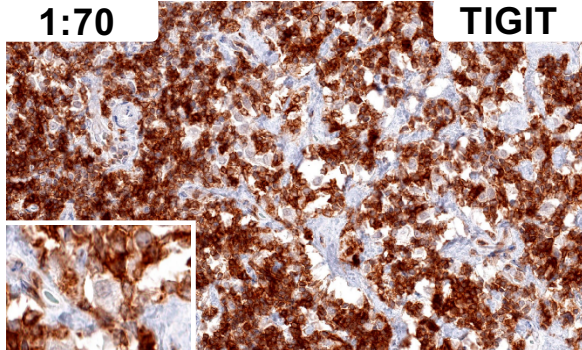

1:210

TIGIT

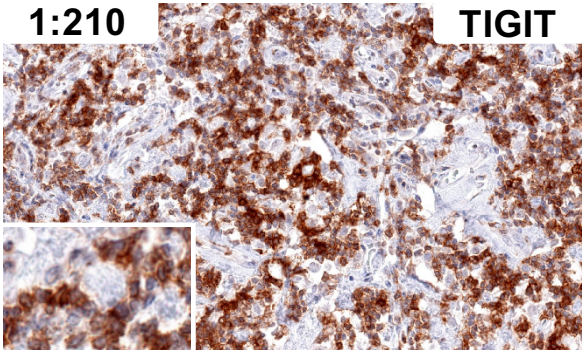

1:630

TIGIT

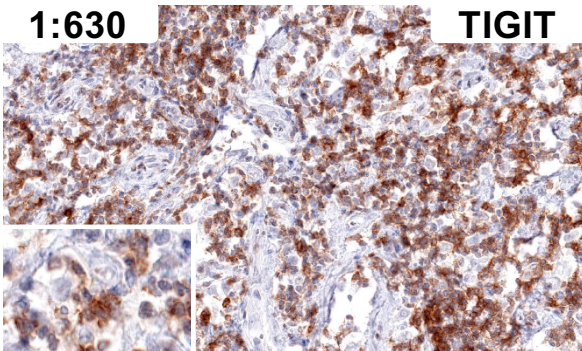

1:1890

TIGIT

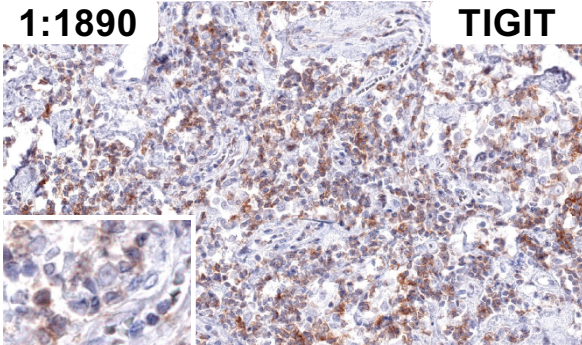

Supplement: Supplementary file 1 — Figure S1. Serial dilution of the TIGIT antibody in lymph node and a NLPHL. (PDF 6352 kb) [file 12885_2018_5111_MOESM1_ESM.pdf]
